# Florence Nightingale

**Published:** 1860-06

**Authors:** 


### FLORENCE NIGHTINGALE.

Florence Nightingale needs but few prefatory remarks. Reared in luxury, and fitted both by natural and acquired endowments to adorn the most elevated social position, her immolation of self and voluntary resignation of those domestic ties which constitute the charm of woman's life, more especially commend her to the world's reverence and praise. Long before the disastrous Crimean war was projected, her gentle ministerings were known throughout Great Britain, Germany, and Egypt, where she attended the sick Arabs. She daily frequented the hospitals and reformatory institutions of London, Edinburgh, and the continent, and, in 1851, went to the great Lutheran Hospital at Kaiserwerth, on the Rhine, where she spent months in constant attendance on the patients, and perfected herself in the art of nursing. On her return to England she undertook the management of the Sanatorium for Governesses, and by her efficient aid, both monetary and personal, saved the institution from the neglect and ruin into which it was fast sinking. Immediately afterwards came accounts of the sufferings in the East, and of the additional hardships which the wounded soldiers were enduring from want of proper care and attention. The English, with their characteristic promptness, soon raised the sum of £15,000, which was expended in articles of food and clothing, for the relief of their army. It was soon felt, however, that the sufferings were chiefly to be ascribed to the absence of proper nursing, and, at the instigation of the Hon. Sydney Herbert, Miss Nightingale was selected as the person best calculated to undertake the control of the British establishment for wounded soldiers and sailors in the Crimea. Many would have shrank from such a proposal, knowing the terrible dangers and toils to which it would necessarily subject them; but Florence Nightingale gloried in the prospect. It was a more extended field for her labors, and one in which her Christian vistues of philanthropy, benevolence, and charity, could be exercised to the utmost.

In October, 1854, accompanied by the Rev. Mr. Bricebridge and his wife, and thirty-seven nurses, she left England on her holy and ennobling mission, reaching Scutari in November, just before the wounded of Balaklava were brought into the hospitals. There it was that she proved herself to be eminently capacitated for the arduous duty she had undertaken. Throughout the wards, where confusion had reigned previous to her arrival, the most perfect order and regularity were maintained; and it was not only while actively engaged for the relief of the sick, that her presence was acknowledge by all to be a blessing. There was many a word of encouragement and smile of sympathy which revived the despondent hearts of the poor sufferers more than any personal aid could possibly have done. In proof of this there is a passage from one of the soldiers' letters, which, though written in a homely style, is replete with poetry which must appeal to every human heart. In speaking of the pleasure it was to see Miss Nightingale pass, he says:

"She would speak to one and to another, and nod and smile to many more, but she couldn't do it to all, you know; we lay there by hundreds; but we could kiss her shadow, as it fell, and lay our heads on the pillows again, content."

After her return from the Crimea, $600,000 were placed at her disposal as a testimony of a nation's gratitude; this sum she appropriated to the erection and management of a Training School for Nurses, thus endeavoring to perpetuate her own example in others.

Very little has been heard of her for two years, but during this time she has not been idle; in her retirement she has written a hand-book called "Notes on Nursing; what it is, and what it is not," which was issued a month ago, in London. This volume is the result of her own experience in her philanthropic career, and by its publication she bestows as great services as she rendered in the hospitals at Scutari.—*N. A. Medico-Chirurgical Review.*

---

Opium an Antidote for Belladonna.—Dr. Lopez, of Mobile, mentions in the *N. A. Medico-Chirurgical Review*, a case of poisoning from belladonna, successfully and speedily relieved by opium, thus showing the complete antagonism of the two poisons. The suggestion was derived from the practice of Dr. Graves, of Dublin. When belladonna is freely used its toxic effects are not uncommon, and it is important to bear in mind this simple antidote.—*St. Louis Med. and Surg. Journal.*

### CHANGES IN MEDICAL SCHOOLS.

New York Medical College: Dr. Horace Green has resigned the presidency of the Faculty of the New York Medical College; and the chairs of Physiology, and of Obstetrics and Diseases of Women and Children, in the same institution, have been vacated by the ritirement of Dr. Austin Flint, Jr., and Dr. E. R. Peaslee.

Dr. W. A. Hammond, U. S. A., has been appointed Professor of Anatomy and Physiology in the University of Maryland, the chair having been resigned by Professor Roby. On account of the death of Professor Frick, the chair of Materia Medica and Therapeutics, in the same college, has been rendered vacant, but has been filled by the appointment of Dr. Edward Warren, of Edenton, North Carolina.

In the New Orleans School of Medicine, a number of changes have been made. Professor Austin Flint, Jr., has been appointed to the chair of Physiology and Microscopic Anatomy, its former incumbent, Dr. Peniston, having been transferred to that of Anatomy. The latter chair was vacated by Professor Beard, who now becomes Professor of Surgery and Surgical Pathology. Another chair, that of Clinical and Operative Surgery, has been established, to which Professor Choppin has been appointed.—*N. A. Medico-Chirurgical Review.*

---

Ointment for Warts.—The *Repertoire de Pharmacie* reproduced from the *Allgemeine Medical Central-Zeitung*, the following prescription for an ointment strongly recommended by Dr. Blaschko for the destruction of warts:

℞ Potassæ chromatis, grs. ij.
Adipis, ℥j. M.

The excrescences should be rubbed with this preparation twice daily, and in the space of three or four weeks the most inveterate verrucose productions are said to be entirely removed.

---

Medical College of the State of South Carolina.—The class in attendance on the lectures for the session of 1859-60, amounted to two hundred and forty-nine students; and the degree of Doctor of Medicine was conferred, at the Annual Commencement, on one hundred and fifteen (115) candidates.

